# Letter to the editor: Regression of liver fibrosis after bariatric operations: A glass two‐third full rather than half empty

**DOI:** 10.1002/hep.32516

**Published:** 2022-04-29

**Authors:** François Pattou, Guillaume Lasailly, Violeta Raverdy, Robert Caiazzo, Philippe Mathurin

**Affiliations:** ^1^ University of Lille, European Genomic Institute for Diabetes Lille France; ^2^ Institut Pasteur Lille Lille France; ^3^ INSERM, UMR 1190, Translational Research for Diabetes Lille France; ^4^ CHU Lille, Integrated Center for Obesity Lille France; ^5^ CHU Lille, General and Endocrine Surgery Lille France; ^6^ Univ. Lille, Inserm, CHU Lille, U1286 ‐ INFINITE ‐ Institute for Translational Research in Inflammation Lille France

To the editor,

We read with interest the report by Pais et al.^[^ ^1^ ^]^ of a series of 66 patients with severe obesity and advanced fatty liver disease proven by liver biopsy during bariatric operations, who subsequently underwent a second biopsy, one to eleven years later. The authors conclude that an operation is highly efficient in improving steatohepatitis, but not to reverse advanced fibrosis, a validated endpoint for evaluating NASH treatment.

In Lassaillly et al.^[^ ^2^ ^]^, we previously reported the improvement of liver fibrosis associated with NASH following bariatric operations.^[^ ^2^ ^]^ In that prospective study, 58 out of 180 participants (32.2% [95% CI, 25.4–39.0]) also had advanced liver fibrosis (≥F3, bridging fibrosis) at baseline, as compared with 71 out of 196 participants in the Pais et al. study (36.2% [95% CI, 29.5–42.9]). Postoperative biopsies were planned after one and five years and completed by more than two‐thirds of eligible participants: 125 out of 169 (74% [95% CI, 67.6–80.6]) at one year, and 64 of 94 patients (68% [95% CI, 58.6–77.4]) at five years. Furthermore, there was no significant difference between the characteristics of patients who underwent repeated liver biopsies and those who did not. Five years after operation, fibrosis decreased in 40 of 57 patients (70.2% [95% CI, 81.6–56.6]), with a resolution of bridging fibrosis in 15 out of 22 patients (68.2% [95% CI, 49.4–88.2]) (Figure 1), and remained stable in 17 patients (26.6% [95% CI, 15.8–37.4]). Thus, fibrosis progressed in only six patients (9.8% [95% CI, 2.5–17.1]), all with persisting NASH. Overall, our findings unveiled the added value of getting rid of NASH, providing direct evidence that advanced fibrosis could be gradually reversed after removing the causal injury.

**FIGURE 1:**
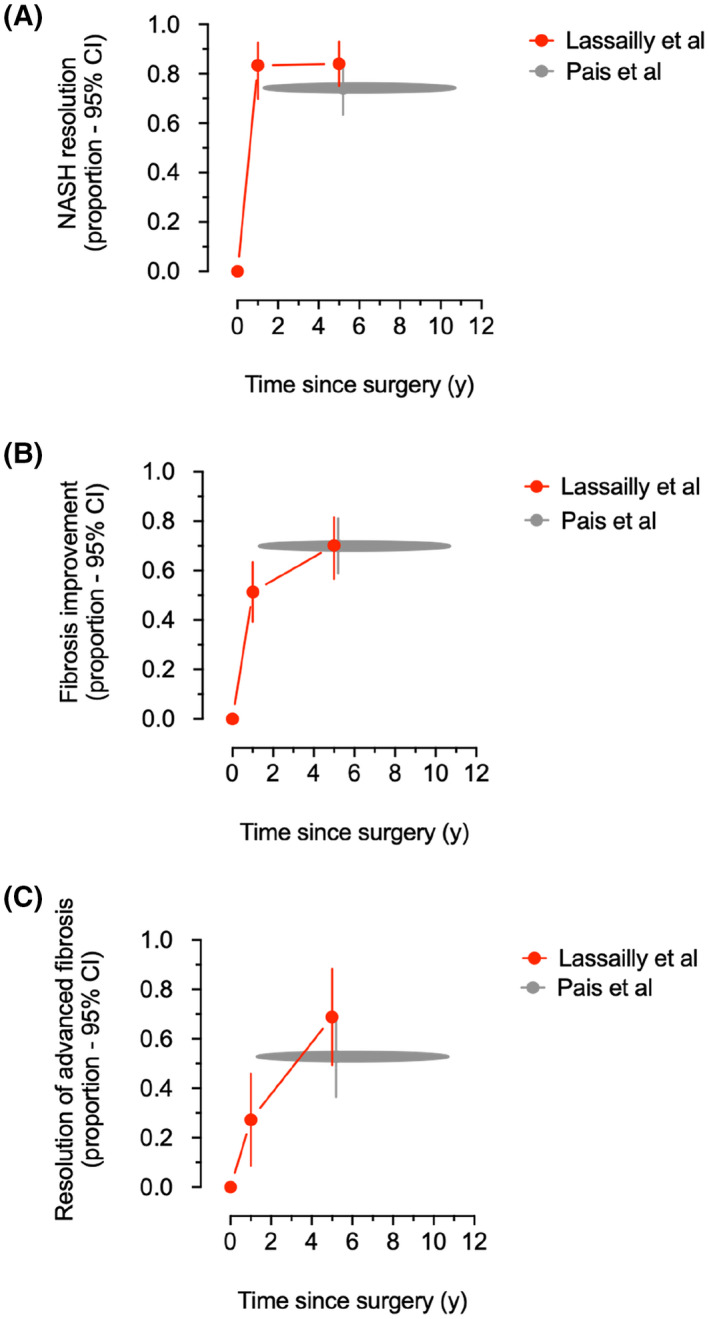
Estimates with 95% confidence intervals (error bars) of the proportions of participants with advanced fatty liver disease who experienced (A) NASH resolution, (B) improvement of liver fibrosis, and (C) resolution of advanced (bridging) fibrosis after bariatric operations at 1 and 5 years in Lassailly et al.^[^ ^2^ ^]^ and between 1 and 11 years in Pais et al.^[^ ^1^ ^]^

In contrast, Pais et al.^[^ ^1^ ^]^ obtained a single postoperative biopsy at variable time points in each patient. Because the length of follow‐up was a major independent predictor of fibrotic response, the authors’ conclusion could have been impacted if the results had been analyzed separately before and after five years. In addition, only one‐third of eligible study participants (66 of 196 patients; 33.8% [95% CI, 27.2–40.4]) underwent repeated biopsy. These individuals were five years older and had more frequent bridging fibrosis and higher plasma aspartate aminotransferase at baseline, suggesting a selection bias favoring patients with more advanced liver disease. However, when the difference in postoperative time is taken into account, data from Pais et al.^[^ ^1^ ^]^ appear to be globally consistent with our prospective results^[^ ^2^ ^]^ (Figure 1).

Altogether, this suggests that the resolution of NASH with operations yields a two‐thirds improvement of liver fibrosis, including bridging fibrosis, over five years or more, and thus outperforms the outcome of pharmaceutical^[^ ^3^ ^]^ or lifestyle^[^ ^4^ ^]^ interventions. Noteworthy, a recent study^[^ ^5^ ^]^ showed that this remarkable outcome through operations could reduce the major liver and adverse cardiovascular outcomes. The rigorous evaluation of surgical interventions for the treatment of advanced NASH in randomized controlled trials is underway (ClinicalTrials.gov Identifier: NCT03472157).

## CONFLICT OF INTEREST

FP is the recipient of a grant from Agence National de la Recherche (Precinash ANR‐16‐RHUS‐006).
